# Feeding habits and habitat use of barking deer (*Muntiacus vaginalis*) in Himalayan foothills, Pakistan

**DOI:** 10.1371/journal.pone.0245279

**Published:** 2021-01-15

**Authors:** Ume Habiba, Maqsood Anwar, Rukhsana Khatoon, Majid Hussain, Kamal Ahmed Khan, Sangam Khalil, Syeda Asma Bano, Ahmed Hussain

**Affiliations:** 1 Department of Wildlife Management, PMAS, Arid Agriculture University Rawalpindi, Rawalpindi, Pakistan; 2 Department of Forestry and Wildlife Management, University of Haripur, Haripur, Pakistan; 3 College of Life sciences, Shandong Normal University, Jinan, China; 4 Department of Forestry Range and Wildlife Management, The Islamia University Bahawalpur, Bahawalpur, Pakistan; 5 Department of Microbiology, University of Haripur, Haripur, Pakistan; Sichuan University, CHINA

## Abstract

Northern red muntjac (*Muntiacus vaginalis*; “barking deer”) is a shy and small-sized cervid mammal, limited to the outer Himalayan foothill forests in Pakistan. Habitat characteristics were measured by locating direct and indirect signs. To quantify habitat utilization of barking deer, 80 field surveys were conducted in the study area along transects. 1200 Quadrats at 50 m intervals were deployed along these transect lines to determine microhabitat factors associated with seasonal distribution. The food composition of the barking deer was determined through fecal droppings analysis by micro-histological technique. Forty-five fecal samples of barking deer were collected from the study area (Murree-Kotli Sattian-Kahuta National Pak); summer (28) and winter (17). The micro-histological analysis revealed that more plant species are available in its habitat during the summer season (27) as compared to winter (19). Due to browsing nature barking deer mostly feed on trees in both seasons. While shrubs are slightly higher in winters. In summer barking deer consumed 10 Trees, 6 Shrubs, 5 Herbs, and 6 kinds of grass species. Dominant tree species were *Phyllanthus emblica* and *Acacia modesta*. Dominant shrub species were *Ziziphus nummularia* and *Justicia adhatoda*. In winter barking deer consumed 8 Trees, 7 Shrubs, 3 Herbs, and 1 Grass. Dominant tree species were *Bauhinia variegata* and *Acacia modesta* while shrubs included *Ziziphus nummularia* and *Carissa opaca*.

## Introduction

Understanding the spatial distributions of wildlife species is a fundamental step in distinguishing the linkages between animals and their potential impacts on natural resources [1, 2]. The choice of ungulate habitat is strongly influenced by nutrients and energy demands for the growth of bones and body mass in males [3] as well as gestation and lactation of calves in females [4]. These body needs are mitigated by the risk of predation, which varies in each habitat type [5]. Energy demands in different genders, particularly the risk of predation [6], have led to multiple examples of seasonal and sexual changes in habitat [7].

Habitat heterogeneity is believed to be important for several reasons. If less of the two resources are required when taken together (e.g. food and cover, or different nutrients), then these resources are said to be complementary [8]. Field observation has shown that this deer consumes fresh, tender fruit and buds and leaves from a variety of low-growing plants [9]. In Thailand, it is particularly fond of the fallen fruit and browses more than he grazes [10]. An opportunistic eater and its stomach contents consisted of a relatively large amount of plants grown on the ground, indicating that it was a selective food that survived as much on grasses [11].

Barking deer is classified as an omnivore and is considered both browser and grazer with grass, ivy, thorny bushes, low leaves, barks, twigs, grasses, fruits, eggs, and small blood animals. Barking deer is usually found at the edge of the forest or in open areas. Barking deer in southern India is also found in large tea plantations because they feed mainly on tea seeds. Their large canines help with the process of food recovery and ingestion [12].

Usually, they feed alone or in two or three, more are usually individuals sharing a food resource. The height of browsing varies between 50 cm and 90 cm but can be up to 20 cm when they stand on their hind legs [13]. Barking deer are nocturnal in their food activity, but can feed at dusk in undisturbed areas. They are very shy and stay close to their area. They defecate elongated pellets with sharp ends, especially places in their potential habitats [14].

General observations on the behavior of Barking Deer were made by Barrette [15] and Lekagul & McNeely [10]. Feeding Barking Deer usually move very slowly, zigzagging over a small area for a long time, usually keeping their nose very close to the ground, stirring the thin litter that covers the surface. Dinerstein [16] described that sometimes, Barking Deer stops for one or two seconds, stretch out his tongue to pick up something on the ground, chew it quickly while keeping the nose close to the ground, and move forward for further exploration.

The drinking behavior of Barking Deer has been described by Barrette [15]. Typically takes water in the middle of the morning and usually chooses a water point as close as possible to the point where he emerges from the forest. Roberts [14] described that he is constantly vigilant during drinking, raising the head every 8–12 seconds.

Barking Deer are considered delicate selective eaters, but seem able to survive as much as grasses. They are particularly similar to ripe fruit falling from various wild figs (*Ficus spp*.), fleshy flowers of silk cotton *(Bombax malabaricum*), and the laurel fruit (*Z*. *mauritiana*), all of which grow in the Margalla ravines frequented by this deer [14].

### Significance of the study

Understanding the effects of barking deer on the forest, feeding habits in the study area is necessary. No quantitative study on the feeding habits of barking deer in Murree, Kotli Sattian, and Kahuta National Park has been conducted. However, the only information on feeding and feeding habits of the barking deer is based on general observations in the field. Not any literature is available on the composition of the diet and its variation with time and space in the Murree, Kotli Sattian, and Kahuta National Park. The present study was therefore conducted to study the seasonal variation in barking deer diet composition.

## Materials and methods

As we didn’t handle or capture the barking deer from the field or study area only the habitat and other parameters of habitat were studies therefore there is no need to get any permission letter from any of the organization along with the field data collection. The consult department staff were also accompanying therefore no illegal act was performed during the field only handling and capturing need permission letter. No disturbance in the core habitat of the species was done.

### Description of the study area

This study was conducted in newly established Murree, Kotli Sattian, and Kahuta National Park located in district Rawalpindi comprising an area of 5,7581 ha. This district is located on the southern slopes of the north-western reaches of the Himalayas, including large expanses of mountains with rich valleys crossed by mountains and rivers. Rawalpindi’s climate has generally been known to change rapidly because of its closeness to the Himalayas and the Pir Panjal Range.

### Data collection

**Fig 1:**
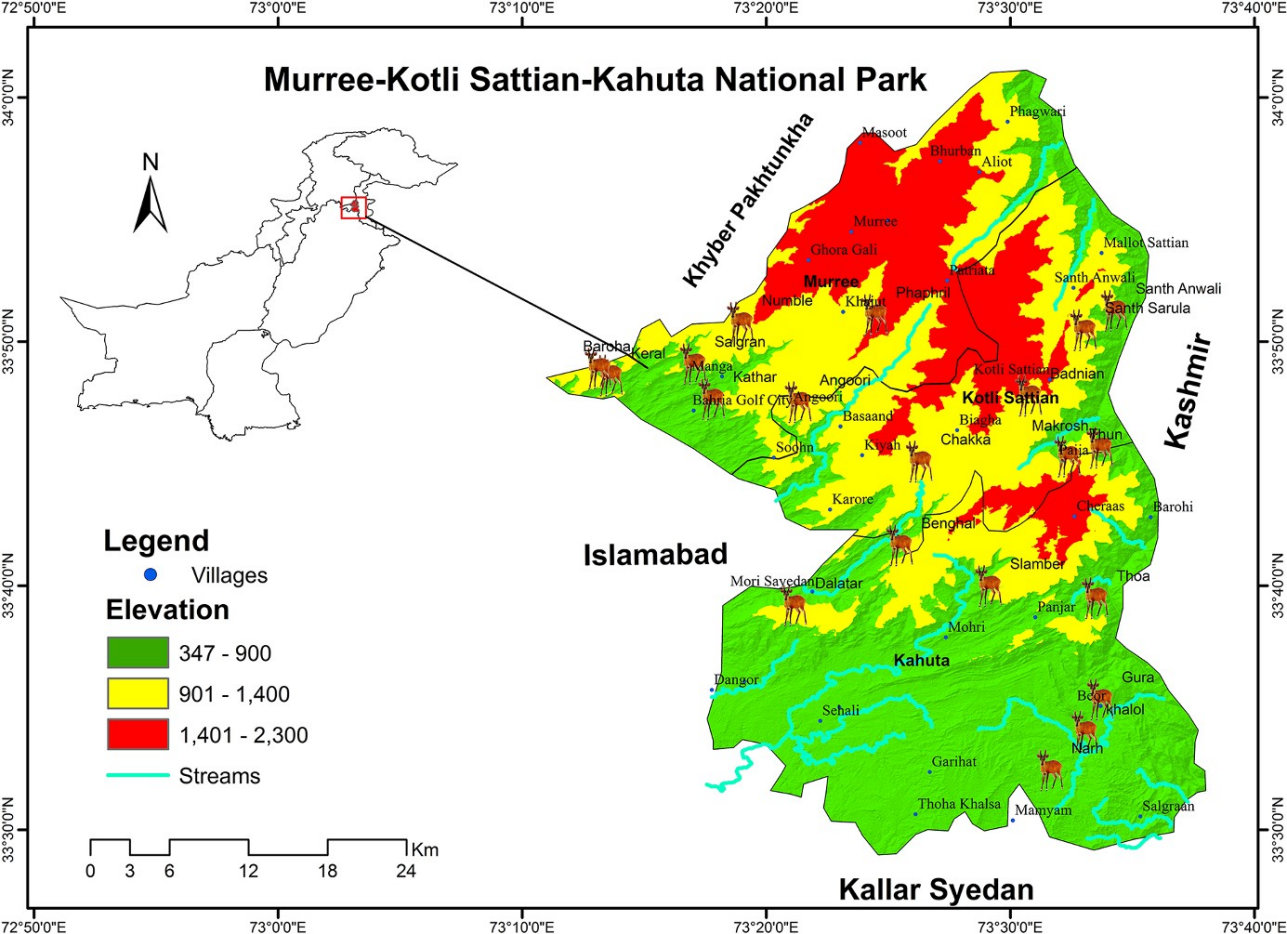
Map of the study area showing study sites of barking deer.

**Fig 2:**
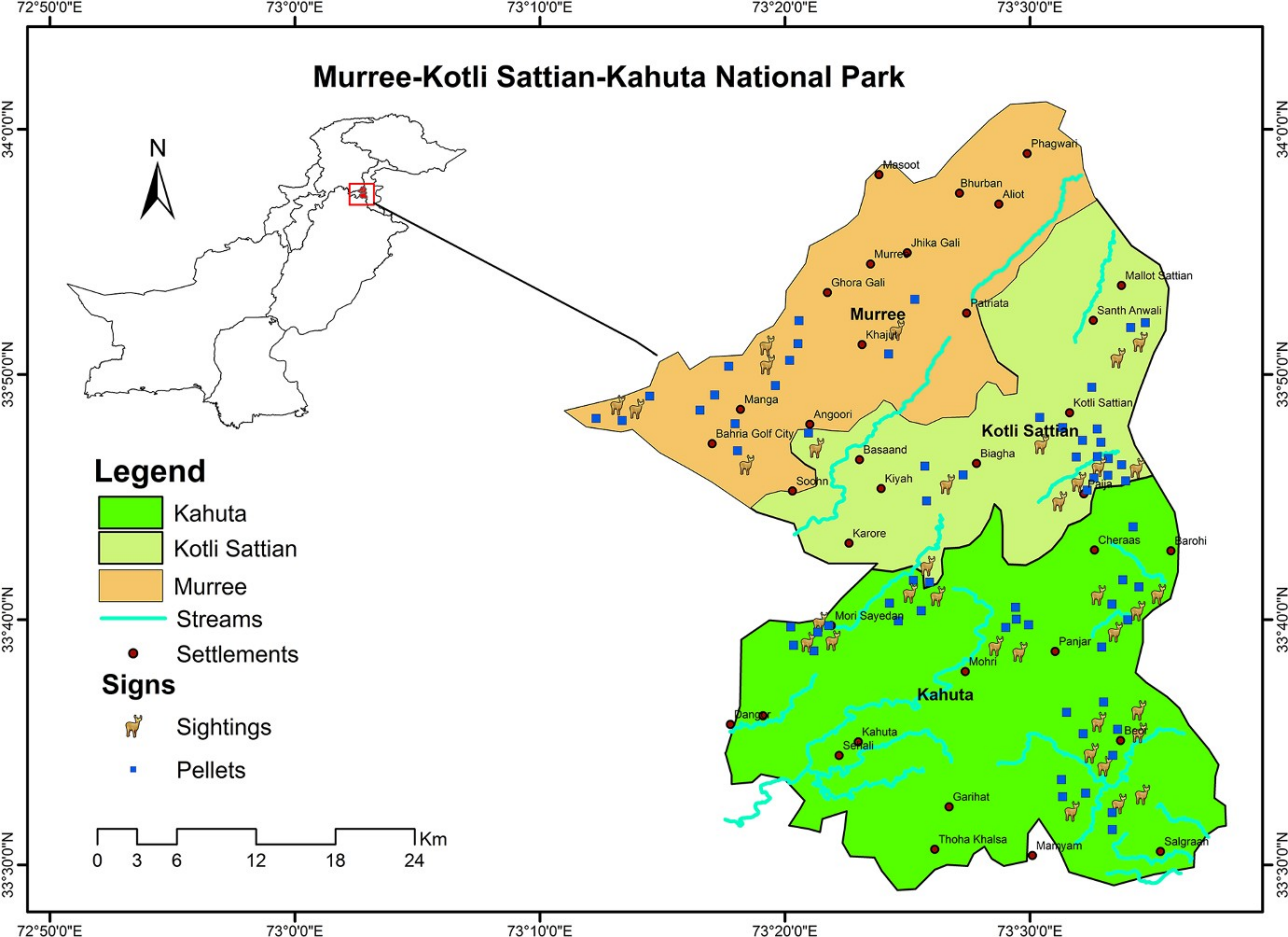
Distribution map of barking deer in Murree, Kotli Sattian and Kahuta National Park.

A reconnaissance survey of the study area, i.e. Murree, Kotli Sattian, and Kahuta National Park was conducted and potential habitat areas of barking deer were identified. Barking deer was found distributed in twenty-four (24) study sites which were further considered as a broad sampling unit for purpose of the present study (Fig 1). The presence was confirmed by direct observation of the barking deer or indirect evidence (footprints, calls, and fecal pellets). Each study site having reasonably uniform physio-biotic conditions was extensively searched by walking through the forest area (Table 1). Elevation and coordinates of barking deer occurrence sites were noted to use for developing a distribution map. The map was developed by using ArcGIS software 10.3 versions (Fig 2).

**Table 1:** Characteristics of study sites in Murree, Kotli Sattian, and Kahuta NP Pakistan.

Site No.	Site name	Location		Elevation (m)	Slope (%)
		North	East		
1	Kathar	33.789677	73.29900123	735–785	20–30
2	Baroha	33.79889627	73.22007127	498–928	50–55
3	Benghal	33.69403773	73.42551447	728–923	70–80
4	Salgran	33.82647088	73.29059175	814–1229	40
5	Angoori	33.79181875	73.352729	798–1143	40–50
6	Numble	33.84065123	73.32231038	1160–1263	60
7	Simli	33.85787864	73.32660107	1073–1263	50
8	Phaphril	33.86014256	73.407826	798–1263	70
9	Gura	33.59281476	73.55842247	557–593	60–70
10	Thoa	33.675747	73.5657615	452–960	40–50
11	Slamber	33.65923889	73.48762222	630–852	30
12	Keral	33.79197273	73.22409836	580–899	50
13	Dalatar	33.657514	73.35559725	740–975	80
14	Beor	33.59296021	73.55634746	538–654	40
15	Seri	33.54116414	73.56057514	545–575	60
16	Sang	33.69057021	73.42647486	538–618	60
17	khalol	33.57534213	73.55849106	547–745	20–30
18	Narh	33.54092813	73.52388563	547–745	30–40
19	Badnian	33.79432177	73.52552046	835–1250	60
20	Makrosh	33.77104682	73.55702127	598–1061	40
21	Thun	33.75810145	73.54085918	977–1120	70
22	Santh Sarula	33.842698	73.55469822	994–1277	60
23	Santh Anwali	33.85818907	73.5751766	663–951	70
24	Chakka	33.75858256	73.44178306	735–786	30

### Phyto-habitat analysis

To quantify the habitat utilization of barking deer, each selected study site was sampled for floral diversity and community structure by quantifying different trees, shrubs, herbs, and grass species during 2015–2017. The quadrat method along a transect line was adopted to determine the seasonal distribution of barking deer during summer (May-October) and winter (November-April). Vegetation data were collected twice a year, once in summer and winter.

Fifty (50) quadrats 50 m apart were taken at each study site, so a total of 1200 quadrats in total were placed perpendicular along a straight line at each sampling point. Density, frequency, and percent cover were noted at each quadrate for each plant species. The size of the sample plot for trees (10m x 10m), shrubs (4m x 4m), grasses, and grass (1m x 1m) were selected [17]. Collected plant samples were pressed, dried, and mounted on herbarium leaves. These plants were identified with already identified plants in the herbarium of Quaid-i-Azam University, Islamabad. Local people were asked to obtain vernacular names of species. To calculate density, frequency, coverage, relative density, relative frequency, and relative coverage equations of [18] were followed.

### Microhistological analysis

The fecal analysis method has been widely adopted to reveal the feeding habits of ungulates [19]. Microhistological analysis of feces is widely used to study the diet composition of a variety of wild and domestic animals from herbivores, such as mammals chewing and degrading plants. The basic principle of this method is that the epidermis cuticle of the plant is a non-digestive part and can be identified by comparable plant reference materials [20]. By this method, the frequency of occurrence of different plant species in the fecal samples was determined [21, 22].

### Sample size

**Fig 3:**
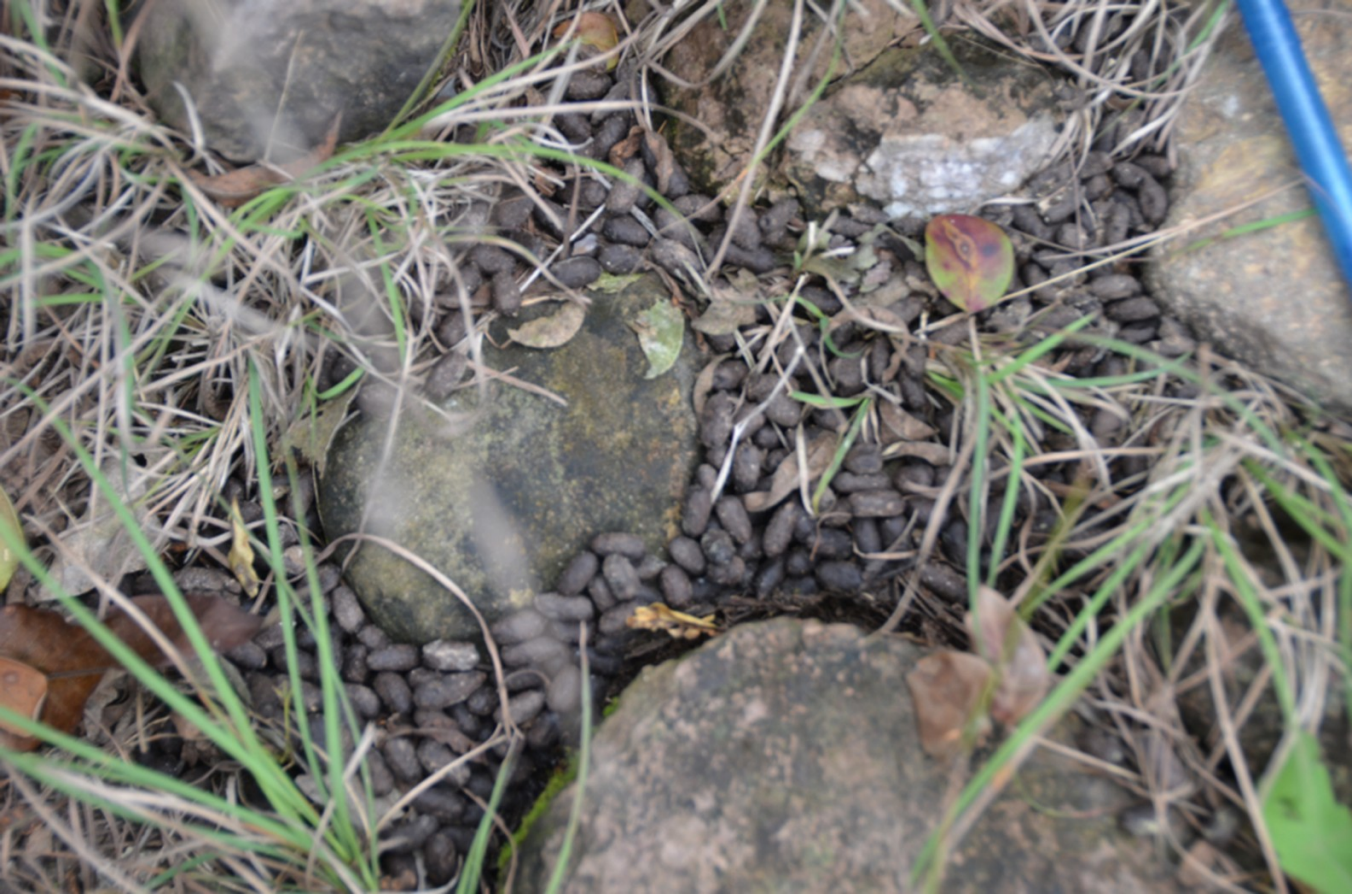
Fecal pellets of barking deer recorded in the study area.

A total of 45 groups of fecal pellets were collected in the study area between August 2015 and November 2017. A group of pellets was considered a sample. Samples were identified by size, structure, and shape (Fig 3).

### Sampling procedure

Fecal samples were collected in summer (28) and winter (17) from twenty-four selected sites along the transect line. The freshness of fecal pellets was determined by color, moisture, and consistency.

### Handling, processing, and storage of fecal pellets

All the collected pellet groups were packed individually in plastic bags, tied with a label containing all information like coordinates, time of collection, and place. The information on the label was recorded using a waterproof pen to minimize the risk of vanishing. The fecal pellets were transported to the Wildlife Management Research Laboratory at the PMAS Arid Agriculture University in Rawalpindi. The samples were air-dried and stored in a dry place.

### Collection of reference plants

The collection of potential species of deer forage was conducted at the same time as the collection of fecal samples at each location for the reference slides. This collection of reference plants was based on information gathered from wildlife personnel, residents in the vicinity of Murree, Kotli Sattian, and Kahuta National Park, and direct observation of grazed vegetation. Two specimens of each plant were collected; one for the reference record and the other for the preparation of the reference key.

### Slide preparation

In the Natural Science Research Laboratory of Wildlife Biology, University of Montana, each sample was ground separately in a mortar and pestle. The material was screened through double folds of fabric to remove dust and unidentifiable particles. This mash was washed in water and soaked in a solution (1 part distilled water, 1 part ethyl alcohol, 1 part glycerin) overnight and then homogenized again in the soil. Each sample is transferred to a test tube containing a freshly prepared solution of 5% sodium hydroxide. The test tube was warm in 5–7 minutes and allowed to precipitate. After decantation, the supernatant was removed. This step was repeated 3–5 times to get a clear liquid.

The material was heated and dehydrated by 25%, 50% to 75%, and 100% alcohol treatments for ten minutes. The alcohol was removed by a series of mixtures of xylene and alcohol. Then, a suitable part of this material was spread on a glass slide and mounted with DPX, and covered with a coverslip. The same procedure was followed by the preparation of the reference plant slides except using a 10% sodium hydroxide solution.

### Slide interpretation

Two slides were prepared from each fecal sample for the microstructure of plant tissues such as cells, stomata fibers, and microscopic spaces. Photomicrographs of the reference plants were taken connected by digital cameras (DCE-2) with a compound microscope (XSZ-701AN / XSZ-107AN). Reference slide micrographs were used to compare and identify materials by Barking Deer with slides prepared from fecal samples.

The presence of plant species in each of the 45 groups of fecal pellets was estimated using an ocular grid micrometer in the microscope eyepiece. Ten micro-fields were selected on each slide and examined. First, the total number of plant species present in the entire slide has been identified. Next, the number of squares of the eye grid is filled with plant material in each row and squares of numbers in each row containing each plant listened to, counted. All data has been tabulated.

The frequency of the different food products of each fecal sample was calculated using the following formula:

Frequencyoccurrence=A/Bx100

Where A is the No. of rows (ocular grid) filled with specific plant species and B is the No. of rows (ocular grid) filled with total plant species identified on the sample slide. This formula is used in two different research articles to determine frequency occurrence or percent composition [20, 22].

Physical characteristics of the habitat at each sampling point, like terrain, slope, water points, and disturbance level were also noted. Elevation and aspect were also noted using Global Positioning System (GPS) and Marching compass. Vegetation data were collected twice a year, once in summer and winter.

## Results

### Phytosociology

Data on the distribution of vegetation cover contributed by different species in different potential habitat areas suggested that a total of 67 plant species (trees = 23, shrubs = 17, herbs = 14, and grasses = 13) were recorded in the habitat of barking deer (Table 2). The tree canopy was open and provided an average of 30 percent vegetative cover.

**Table 2:** Analysis of plant species found in barking deer habitat.

S.No.	Plant Species	Density	Frequency	Cover	Relative Density	Relative Frequency	Relative cover	Importance Value
**Tree**								
1	*Acacia catechu*	0.3	2.4	0.7	0.19	2.7	0.71	3.6
2	*Acacia modesta*	2.4	34.7	2.8	1.55	39.08	2.84	43.47
3	*Acacia nilotica*	1.5	10.4	0.8	0.97	11.71	0.81	13.49
4	*Albizia lebbeck*	0.7	14.6	1.2	0.45	16.44	1.22	18.11
5	*Albizia odoratissima*	0.1	1.3	0.4	0.06	1.46	0.41	1.93
6	*Bauhinia varigata*	0.6	5.6	0.4	0.39	6.31	0.41	7.11
7	*Celtis australis*	1.3	19.8	1.1	0.84	22.3	1.12	24.26
8	*Dalbergia sissoo*	1.8	16.8	1.2	1.16	18.92	1.22	21.3
9	*Ficus bipinnata*	0.1	3.2	0.4	0.06	3.6	0.41	4.07
10	*Flacourtia indica*	1.1	24.5	1.4	0.71	27.59	1.42	29.72
11	*Grewia optiva*	0.3	5.1	0.6	0.19	5.74	0.61	6.54
12	*Mallotus philippinensis*	2.1	34.4	2.8	0.01	38.74	2.84	41.59
13	*Olea ferruginea*	1.5	41.2	2.3	0.97	46.4	2.33	49.7
14	*Phyllanthus emblica*	0.9	5.3	1.8	0.58	5.97	1.83	8.38
15	*Pinus roxburghii*	1.2	42.4	12.7	0.78	47.75	12.89	61.42
16	*Pinus wallichiana*	2.7	31.2	3.1	1.75	35.14	3.15	40.04
17	*Phoenix loureiri*	0.5	4.3	1.2	0.32	4.84	1.22	6.38
18	*Pistacia chinensis*	0.1	2.3	1.4	0.06	2.59	1.42	4.07
19	*Pyrus pashia*	0.6	7.1	0.3	0.39	7.1	0.3	7.79
20	*Quercus incana*	1.6	18.7	3.0	1.03	21.06	3.05	25.14
21	*Syzygium cumini*	0.1	2.3	1.4	0.06	2.59	1.42	4.07
22	*Xylosma longifolia*	1.9	17.3	0.8	1.23	19.48	0.81	21.52
23	*Ziziphus mauritiana*	0.5`	6.2	0.7	0.19	2.7	0.71	3.6
**Shrubs**								
1	*Berberis lyceum*	0.3	2.7	1.5	0.19	3.04	1.52	4.75
2	*Carissa opaca*	21.8	87.4	32.3	14.1	98.42	32.8	145.32
3	*Deutzia staminea*	0.7	4.2	0.9	0.45	4.73	0.91	6.09
4	*Dodonaea viscosa*	15.6	76.7	20.0	10.1	87.37	20.3	117.77
5	*Galium asperifolium*	0.2	2.4	1.3	0.13	2.7	1.32	4.15
6	*Justicia adhatoda*	5.7	29.8	2.1	3.69	33.56	2.13	39.38
7	*Lantana camara*	1.3	12.7	2.0	0.84	14.3	2.03	17.17
8	*Lespedeza cuneata*	4.3	43.6	1.2	2.78	49.1	1.22	53.1
9	*Maytenus royleanus*	3.1	73.2	2.4	2.01	82.43	2.44	86.88
10	*Myrsine africana*	6.7	61.4	14.5	4.33	69.14	14.72	88.19
11	*Nerium oleander*	1.7	14.2	0.9	1.1	15.1	0.91	17.11
12	*Puncia granatum*	1.7	36.8	0.8	1.1	41.44	0.81	43.35
13	*Rosa webbiana*	0.6	2.8	1.7	0.39	3.15	1.73	5.27
14	*Rubus ellipticus*	5.2	32.4	11.5	3.36	36.49	11.68	51.53
15	*Sarcococca saligna*	0.4	2.2	1.3	0.26	2.48	1.32	4.06
16	*Woodfordia fruticosa*	4.4	53.8	2.7	2.85	60.59	2.74	66.18
17	*Ziziphus nummularia*	0.2	1.8	0.7	0.13	2.03	0.71	2.87
**Herbs**								
1	*Adiantum incisum*	5.2	18.9	2.2	3.36	21.28	2.23	26.87
2	*Barleria cristata*	0.4	9.6	0.2	0.29	10.81	0.2	11.3
3	*Diclyptera bupleuroides*	2.3	4.5	0.8	1.49	5.068	0.81	7.37
4	*Duchesnea indica*	2.7	5.5	0.4	1.75	6.19	0.41	8.35
5	*Euphorbia aucheri*	5.6	18.9	1.7	3.62	21.28	1.73	26.63
6	*Indigofera linifolia*	1.7	3.5	0.8	1.1	3.94	0.81	5.85
7	*Launaea procumbens*	0.4	7.8	0.1	0.26	8.78	0.1	9.14
8	*Lespedeza floribunda*	0.1	1.8	0.4	0.06	2.03	0.41	2.5
9	*Lespedeza juncea*	0.3	1.2	0.6	0.19	1.35	0.61	2.15
10	*Micromeria biflora*	2.8	39.7	0.6	1.81	44.71	0.61	47.13
11	*Oxalis corniculata*	7.6	23.9	1.4	4.92	26.91	1.42	33.25
12	*Saussurea heteromella*	7.3	27.6	0.9	4.72	31.08	0.91	36.71
13	*Sida cordifolia*	3.1	47.3	1.2	2.01	53.27	1.22	56.5
14	*Thalictrum foetidum*	1.2	5.6	0.3	0.78	6.31	0.3	7.39
**Grasses**								
1	*Apluda mutica*	2.1	17.3	1.6	1.36	19.48	1.62	22.46
2	*Aristida mutabilis*	1.7	0.5	0.3	1.1	0.56	0.3	1.96
3	*Bothriochloa ischaemum*	1.6	9.1	1.5	1.03	10.25	1.52	12.8
4	*Brachiaria ramose*	0.3	0.2	0.1	0.2	0.22	0.1	0.52
5	*Cymbopogon jwarancusa*	3.4	12.3	2.6	2.2	13.85	2.64	18.69
6	*Cynodon dactylon*	1.9	10.3	1.4	1.23	11.6	1.42	14.25
7	*Desmostachya bipinnata*	0.3	0.2	0	0.2	0.21	0	0.41
8	*Dichanthium annulatum*	1.7	9.3	1.2	1.1	10.47	1.22	12.79
9	*Eleusine indica*	0.3	0.1	0	0.2	0.11	0	0.31
10	*Heteropogon contortus*	0.4	0.1	0.1	0.26	0.11	0.1	0.47
11	*Imperata cylindrical*	0.8	0.5	0.3	0.52	0.56	0.3	1.38
12	*koeleria macrantha*	0.5	0.7	0.2	0.32	0.79	0.2	1.31
13	*Themeda anathera*	1.1	0.4	0.2	0.71	0.45	0.2	1.36

### Seasonal diet composition

**Fig 4:**
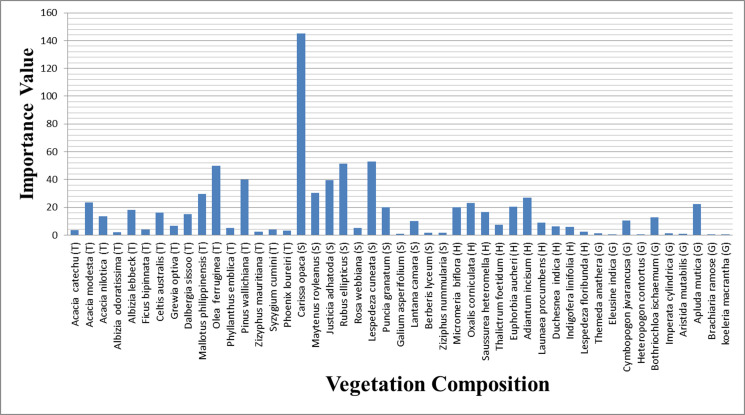
Plant species recorded in summer from the habitat of barking deer.

The summer habitat of barking deer consisted of 47 plant species which comprised 16 tree species, 11 shrub species, 10 herb species, and 10 grass species (Fig 4). Dominant tree species were *Olea ferruginea* (IV = 49.70) and *Pinus wallichiana* (IV = 40.04), dominant shrub species were *Carissa opaca* (IV = 145.32) and *Rubus ellipticus* (IV = 51.53), dominant herb species were *Adiantum incisum* (IV = 26.87) and *Oxalis corniculata* (IV = 23.1) and dominant grass species were *Apluda mutica* (IV = 22.46) and *Bothriochloa ischaemum* (IV = 12.8).

Out of 28 fecal samples collected in the study area in summer, 27 plant species (tree = 10, shrubs = 6, grass = 5, and grass = 6) were recorded in the diet of barking deer. The analysis of fecal pellets revealed that the diet of deer consisted mainly of trees (37.03%), which constituted a major component of their diet, followed by shrubs and grasses consumed (22.22%). Presence of various foods in fecal samples recorded in the following order: *Phyllanthus emblica > Ziziphus nummularia > Justicia adhatoda > Acacia modesta > Grewia optiva > Apluda mutica > Bauhinia variegata > Carissa opaca > Micromeria biflora > Adiantum incisum > Woodfordia fruticosa > Bauhinia variegata > Maytenus royleanus > Bothriochloa ischaemum*. The rest of the plant species were consumed in trace amounts (Table 3).

**Table 3:** Frequency occurrence (%) of different food items in fecal samples (summer season) of barking deer.

S.No.	Vernacular names	Plant species	Part used	% Occurrence
1	Amla	*Phyllanthus emblica (T)*	leaves	57.24
2	Phulai	*Acacia modesta (T)*	leaves	31.56
3	Dhaman	*Grewia optiva (T)*	leaves	27.3
4	Kachnar	*Bauhinia variegata (T)*	leaves	26.35
5	Beri	*Zizyphus mauritiana (T)*	leaves	7.9
6	Kamila	*Mallotus philippinensis (T)*	leaves	6.9
7	Dandal	*Xylosma longifolia (T)*	leaves	4.5
8	Phagwara	*Ficus bipinnata (T)*	leaves	3.8
9	Ban	*Quercus incana (T)*	leaves	1.3
10	Chir pine	*Pinus roxburghii (T)*	leaves	1.2
11	Khair	*Ziziphus nummularia (S)*	leaves	46.2
12	Baiker	*Justicia adhatoda (S)*	leaves	33.6
13	Grinda	*Carissa opaca (S)*	leaves	22.7
14	Dhawi	*Woodfordia fruticosa (S)*	leaves	17.56
15	Pataki	*Maytenus royleanus (S)*	leaves	16.8
16	Gukoon	*Myrsine Africana (S)*	leaves	13.7
17	-	*Adiantum incisum (H)*	leaves	18.5
18	Boine	*Micromeria biflora (H)*	leaves	5.1
19	Khatti buti	*Oxalis corniculata (H)*	leaves	4.16
20	Kali jiri	*Saussurea heteromella (H)*	leaves	3.6
21	Beni	*Thalictrum foetidum (H)*	leaves	2.4
22	Ghagari	*Apluda mutica (G)*	leaves	26.8
23	Palwan	*Bothriochloa ischaemum (G)*	leaves	16.5
24	Khuskus grass	*Cymbopogon jwarancusa (G)*	leaves	9.7
25	Sarriyala gaas	*Heteropogon contortus (G)*	leaves	7.4
26	Mandhano	*Eleusine indica (G)*	leaves	6.8
27	White grass	*Themeda anathera (G)*	leaves	3.8

**Fig 5:**
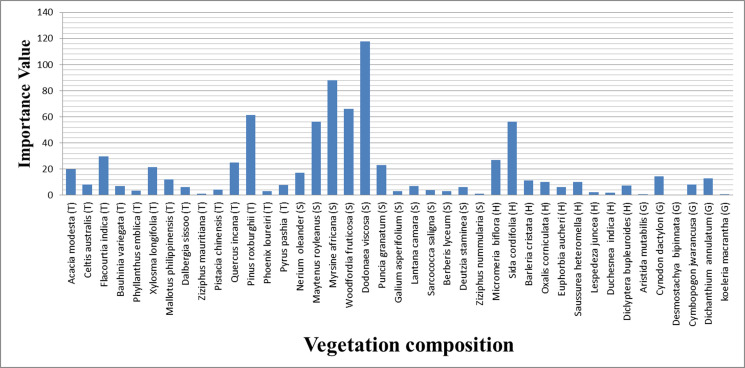
Plant species recorded in winter from the habitat of barking deer.

A total of 41 plant species were observed in the winter habitat of barking deer, which included 14 tree species, 12 shrub species, nine grass species, and six grass species.

(Fig 5) Dominant tree species during winter were *Pinus roxburghii* (IV = 61.42) and *Flacourtia indica* (IV = 29.72), dominant shrub species were *Dodonaea viscosa* (IV = 117.77) and *Myrsine africana* (IV = 88.19), dominant herb species were *Sida cordifolia* (IV = 56.5) and *Micromeria biflora* (IV = 27.05) and dominant grass species were *Cynodon dactylon* (IV = 14.25) and *Dichanthium annulatum* (IV = 12.79).

In winter, a total of 19 plant species (tree = 8, shrubs = 7, grass = 3 and grass = 1) were recorded in the diet of barking deer. Analysis of fecal pellets revealed that diet is predominantly composed of trees (42.10%), followed by shrubs (36.84%) and grasses (15.78%). Only herbs (5.26%) were consumed. Winter is a time when most grasses become dry due to night freezing. The only green matter available to the deer was leaves of shrubs and trees which were a major component of his diet. Presence of various foods in fecal samples recorded in the following order: *Ziziphus nummularia > Oxalis corniculata > Carissa opaca > Bauhinia variegata > Justicia adhatoda > Acacia modesta > Phyllanthus emblica > Puncia granatum > Pinus roxburghii > Grewia optiva*. The rest of the plant species were consumed in trace amounts (Table 4).

**Table 4:** Frequency occurrence (%) of different food items in fecal samples (winter season) of barking deer.

S.No.	Vernacular names	Plant species	Part used	% Occurrence
1	Kachnar	*Bauhinia variegata (T)*	leaves	25.8
2	Phulai	*Acacia modesta (T)*	leaves	19.52
3	Amla	*Phyllanthus emblica (T)*	leaves & fruit	19.04
4	Chir pine	*Pinus roxburghii (T)*	leaves	11.78
5	Dhaman	*Grewia optiva (T)*	leaves	11.7
6	Kahu	*Olea ferruginea (T)*	leaves & fruit	8.4
7	Ban	*Quercus incana (T)*	leaves	3.1
8	Beri	*Zizyphus mauritiana (T)*	leaves	2.7
9	Khair	*Ziziphus nummularia (S)*	leaves & fruit	53.5
10	Grinda	*Carissa opaca (S)*	leaves	32.3
11	Sumbul	*Berberis lyceum (S)*	leaves	29.8
12	Baiker	*Justicia adhatoda (S)*	leaves	24.6
13	Daruna	*Puncia granatum (S)*	leaves	13.44
14	Gukoon	*Myrsine Africana (S)*	leaves	6.1
15	Pataki	*Maytenus royleanus (S)*	leaves	5.3
16	Khatti buti	*Oxalis corniculata (H)*	leaves	42.7
17	Kali jiri	*Saussurea heteromella (H)*	leaves	9.4
18	Dil-patri	*Sida cordifolia (H)*	leaves	2.3
19	Khabal	*Cynodon dactylon (G)*	leaves	6.3

### Distribution of barking deer in the study area

Barking deer was not evenly distributed with vegetation types of the study area. The most preferred vegetation types were trees and shrubs with 30 and 69 percent cover, respectively. Barking deer was mostly distributed in shrubby vegetation as compared to avoided thick tree cover which ultimately hinders free movement and escapes of barking deer from a predator. More fecal pellets were collected near shrub species. No significant difference (χ² = 6.37, df = 3, p = 0.19 < 0.05) in seasonal vegetation cover was recorded in habitat of barking deer.

Comparison of plant species recorded in barking deer diet in two different seasons revealed that a significantly higher plant species (t = 5.78, p = 0.04 < 0.005) were available in summer as compared to winter.

### Diet preference

Analysis of fecal samples of barking deer showed a high preference for trees in both seasons as mostly tree leaves were consumed. Dominant tree species were Phyllanthus emblica, *Acacia modesta*, *Grewia optiva*, *Bauhinia varigata*, and shrubs including *Ziziphus nummularia*, *Justicia adhatoda*, *Carissa opaca*, *Woodfordia fruticosa*, *Maytenus royleanus*, and *Myrsine africana*. Preferred herbal species were *Oxalis corniculata Adiantum incisum*, *Micromeria biflora*, and *Saussurea heteromella*. Only two grass species *Apluda mutica* and *Bothriochloa ischaemum* were recorded in large quantities from fecal samples. The barking deer was recorded as a browser during the winter and it was a browser and a grazer in the summer. There was no sign of his predation on animal matter.

## Discussion

Data on the distribution of vegetation in potential habitat areas suggested that most of the vegetation cover was occupied by shrubs (69%). It avoided thick tree cover which ultimately hinders movement and escapes from a predator. The tree canopy was opened and provides an average of 30 percent of total vegetative cover.

The results of the present study generally support the findings of Hameed et al. [23], who suggested a major part of the absolute cover of barking deer habitat was contributed by shrubs (30.3–68.7%.), tree cover above 20% might be lethal for survival from a predator. In an article, it is reported that barking deer is a browser, so greater shrub cover ensures the availability of food [10]. The preference of the thick canopy of barking deer can reduce visualization which is an anti-predatory strategy [24]. The selection of a high percentage of plant cover is also a predator defense strategy for ungulate calves [25, 26].

Information on food habits of this study suggested that barking deer is a browser as it tends to be feed on leaves of plant species present in the study area. Similar trends were reported in Thailand, that barking deer browses more than it grazes [10].

No seasonal changes in diet were observed and the results of this study revealed that seasonal differences are associated with the percentage of plant species available in each season. Significant seasonal differences in the diet of barking deer were recorded in the frequency of consumption of different foods, but not in terms of volume [27]. The presence of trees, shrubs, and forbs in the diet of barking deer was proportional to their availability in the habitat during both seasons. None of the plant species were used more than its availability [28].

In both seasons trees and shrubs dominated the flora and it contributed a major part of the diet of Barking Deer. In winter low availability of grasses due to frost was avoided in food items. Similar trends were observed in Chitwan National Park [29]. They switched to other food items when grass availability became less in winter. He also observed that the animals used to feed on fresh twigs of *Imperata cylindrica*. The newly growing plant leaves and shoots in the rainy season could cause deer to switch from grazing to browsing.

This study suggests that the barking deer feed mainly on the leaves and fruits of *Phyllanthus emblica*, *Olea ferruginea*, and *Ziziphus nummularia*. While only the leaves of the rest of the plant species were consumed. In Pakistan, barking deer preferred ripened fallen fruits of wild figs (*Ficus* sp.), fleshy flowers of *Salmalia malabarica* (Cymbal), and fruits of *Zizyphus nimmularia* (jangliberi or mallah) [14]. The diet of barking deer in Nepal; he prefers grasses, such as *Imperata cylindrica* and *Cynodon dactylon*, and fallen trees fruits, such as *Ficus glomerata*, *Schleidora trijuga*, and *Eugenia jambolina* [16]. Field observation has shown that this deer consumes fresh, tender fruit and buds and leaves from a variety of growing plants [30]. *Imperata cylindrica*, *Smilax aspera*, *Moghania strobilifera*, and *Arundinari afalcata* were major food items in both seasons. Fruits of *Prunus cerasoides*, *Berberis asiatica*, and *Rubus ellipticus* and bark of *Pinus roxburghii* and *Castanopsis indica* were also taken in significant amounts [27].

In this study, no evidence of animal forage was recorded in the diet of barking deer. No animal remains registered in the diet [27]. The results of the study suggest that barking deer were mixed feeders.

### Management recommendation

Deer species are the best indicator of the forest because they are specialized in habitat and particularly sensitive to habitat changes. Given the food requirements and availability of Barking Deer in different habitats, managers should maintain the strategy in a way that minimizes grazing pressure and anthropogenic pressure and maximizes the efficiency of deer feeding. Also, park management regimes must ensure that the availability of preferred plant species in barking deer habitat exclusively. Similar data on the feeding habits of wild ungulates will be important for the rational management of many protected areas in Pakistan.

## Supporting information

S1 Annex Record of observations in each study site at different field visits. (DOCX)

## References

[1] McShea WJ, Underwood HB, Rappole JH. Science of overabundance Deer Ecology and Population Management. USA: Smithsonian Institution Press 1997.

[2] Liu J, Taylor WW. Coupling landscape ecology with natural resource management: Paradigm shifts and. Integrating Landscape Ecology into Natural Resource Management. 2002(1):3.

[3] Bronson FH. Mammalian reproductive biology. University of Chicago Press; 1989.

[4] Belovsky GE. Generalist herbivore foraging and its role in competitive interactions. American Zoologist. 1986 2 1; 26(1):51–69.

[5] Leslie DM Jr, Bowyer RT, Kie JG. Life-history strategies of ungulates. Journal of Mammalogy. 1999 12 6; 80(4):1067–9.

[6] Kie JG. Optimal foraging and risk of predation: effects on behavior and social structure in ungulates. Journal of Mammalogy. 1999 12 6; 80(4):1114–29.

[7] Main MB, Weckerly FW, Bleich VC. Sexual segregation in ungulates: new directions for research. Journal of Mammalogy. 1996 5 17; 77(2):449–61.

[8] Tilman D. Resource competition and community structure. Princeton university press; 1982 8 21. 7162524

[9] Nagarkoti erwerf A. Udjung Kulon: the land of the last Javan rhinoceros. Brill Archive; 1970.

[10] Lekagul B, McNeely JA. Mammals of Thailand. Bangkok, Thailand: Kurusapa Ladprao Press 1977.

[11] Chaplin RE. Deer. Blandford; 1977.

[12] Grubb P. Wilson DE, Reeder DM, editors. Mammal species of the world: a taxonomic and geographic reference. JHU Press; 2005.

[13] Prior R. Deer Watch (2nd Ed). Deer Identification sheet. British Deer Society 2007.

[14] Roberts TJ, Bernhard (principe d’Olanda.). The mammals of Pakistan 1997.

[15] Barrette C. Some aspects of behavior of muntjacs in Wilpatto National Park. Mammalia. 1977; 41: 1–34.

[16] Dinerstein E. An ecological survey of the Royal Karnali-Bardia Wildlife Reserve, Nepal: part III: ungulate populations. Biological Conservation. 1980 6 1; 18(1):5–37.

[17] Schemnitz SD. Wildlife management techniques manual. Wildlife Society; 1980.

[18] Hussain F. Manual of Plant Ecology. Univ. Grants Commission, Sector H-8, Islamabad, Pakistan; 1983.

[19] Kessler WB, Kasworm WF, Bodie WL. Three methods compared for analysis of pronghorn diets. The Journal of Wildlife Management. 1981 7 1:612–9.

[20] Sparks DR, Malechek JC. Estimating percentage dry weight in diets using a microscopic technique. Rangeland Ecology & Management/Journal of Range Management Archives. 1968 6 30;21(4):264–5.

[21] Holechek JL, Vavra M, Pieper RD. Botanical composition of determination of range herbivore diets. A review Grazing animals, forage resources. Rangeland Ecology & Management/Journal of Range Management Archives. 1982 5 1; 35(3):309–15.

[22] Alipayo DA, Valdez R, Holechek JL, Cardenas M. Evaluation of microhistological analysis for determining ruminant diet botanical composition. Rangeland Ecology & Management/Journal of Range Management Archives. 1992 3 1; 45(2):148–52.

[23] Hameed W., & Mian A. Population features of barking deer (Muntiacus muntjak) in Margalla Hills National Park, Pakistan. Pakistan Journal of Zoology. 2009 4 1; 41(2).

[24] Geist V. On the relationship of social evolution and ecology in ungulates. American zoologist. 1974 2 1; 14(1):205–20.

[25] Gerlach TP, Vaughan MR. Mule deer fawn bed site selection on the pinon canyon maneuver site, Colorado. The Southwestern Naturalist. 1991 6 1; 36(2):255–8.

[26] Bowyer RT, Van Ballenberghe V, Kie JG, Maier JA. Birth-site selection by Alaskan moose: maternal strategies for coping with a risky environment. Journal of Mammalogy. 1999 12 6; 80(4):1070–83.

[27] Nagarkoti A, Thapa T. Food habits of barking deer (Muntiacus muntjac) in the middle hills of Nepal. Hystrix, the Italian Journal of Mammalogy. 2005 7 9; 18(1).

[28] Ilyas O, Khan JA. Food habits of barking deer (Muntiacus muntjak) and goral (Naemorhedus goral) in Binsar Wildlife Sanctuary, India. Mammalia. 2003 1 1; 67(4):521–32.

[29] Yonzon PB. Ecological studies on Muntiacus muntjak (Zimmerman)[barking deer, Nepal]. Journal of Natural History Museum (Nepal). 1978.

[30] Hoogerwerf A. Udjung Kulon: the land of the last Javan rhinoceros. Brill Archive; 1970.

