# Supplementary material for: Feeding habits and habitat use of barking deer (Muntiacus vaginalis) in Himalayan foothills, Pakistan
Source: PLoS One. 2021 Jan 15;16(1):e0245279. doi: 10.1371/journal.pone.0245279 (PMC7810515; doi:10.1371/journal.pone.0245279)
Supplement: S1 Annex — (DOCX) [file pone.0245279.s001.docx]

**S1 Annex. RECORD OF OBERVATIONS IN EACH STUDY SITE AT DIFFERENT FIElD VISITS**

| Name of Tehsil | Study site | Area km^2^ | transect taken at different visits | distance (m) | Record of observation |
| --- | --- | --- | --- | --- | --- |
| Kahuta | Gura | 307 | 1 | 300 | Feacal pellets collected |
|  | Gura |  | 2 | 250 | Foot prints found |
|  | Gura |  | 2 | 500 | one male sighted |
|  | Gura |  | 3 | 350 | Foot prints found |
|  | Soha | 90 | 1 | 100 | Call heard |
|  | Soha |  | 1 | 500 | Foot prints found |
|  | Soha |  | 1 | 200 | Foot prints found |
|  | Soha |  | 1 | 100 | Foot prints found |
|  | Soha |  | 2 | 150 | Call heard |
|  | Soha |  | 2 | 500 | one female with one fawn sighted |
|  | Soha |  | 2 | 100 | Foot prints found |
|  | Soha |  | 2 | 200 | Feacal pellets collected |
|  | Soha |  | 2 | 100 | one male sighted |
|  | Soha |  | 2 | 650 | one female with one fawn sighted |
|  | Soha |  | 3 | 100 | Foot prints found |
|  | Soha |  | 3 | 300 | Feacal pellets collected |
|  | Slamber | 211 | 1 | 100 | Call heard |
|  | Slamber |  | 1 | 300 | Foot prints found |
|  | Slamber |  | 2 | 500 | one male sighted |
|  | Slamber |  | 2 | 100 | Foot prints found |
|  | Slamber |  | 3 | 100 | Feacal pellets collected |
|  | Slamber |  | 3 | 100 | Feacal pellets collected |
|  | Keral | 2192 | 1 | 500 | one female with one fawn sighted |
|  | Keral |  | 1 | 100 | Feacal pellets collected |
|  | Keral |  | 1 | 100 | Feacal pellets collected |
|  | Keral |  | 2 | 200 | Call heard |
|  | Keral |  | 3 | 350 | one male sighted |
|  | Keral |  | 3 | 400 | Foot prints found |
|  | Dalatar | 307 | 1 | 200 | Feacal pellets collected |
|  | Dalatar |  | 1 | 200 | Feacal pellets collected |
|  | Dalatar |  | 1 | 200 | Foot prints found |
|  | Dalatar |  | 2 | 200 | Feacal pellets collected |
|  | Dalatar |  | 2 | 500 | Call heard |
|  | Dalatar |  | 2 | 700 | one male sighted |
|  | Dalatar |  | 2 | 250 | Call heard |
|  | Dalatar |  | 3 | 100 | Foot prints found |
|  | Dalatar |  | 3 | 1000 | one female sighted |
|  | Dalatar |  | 3 | 100 | Feacal pellets collected |
|  | Beor | 40 | 1 | 100 | Feacal pellets collected |
|  | Beor |  | 2 | 100 | Foot prints found |
|  | Beor |  | 2 | 900 | one male sighted |
|  | Beor |  | 3 | 200 | Feacal pellets collected |
|  | Seri | 182 | 2 | 400 | Call heard |
|  | Seri |  | 2 | 400 | one pair found |
|  | Seri |  | 3 | 200 | Feacal pellets collected |
|  | Seri |  | 3 | 200 | Foot prints found |
|  | Sang | 1374 | 1 | 200 | Foot prints found |
|  | Sang |  | 2 | 600 | a pair sighted |
|  | Sang |  | 2 | 100 | Foot prints found |
|  | Sang |  | 3 | 100 | Feacal pellets collected |
|  | khalol | 43 | 1 | 300 | Call heard |
|  | khalol |  | 1 | 500 | 2 females sighted |
|  | khalol |  | 2 | 300 | Foot prints found |
|  | khalol |  | 2 | 300 | Feacal pellets collected |
|  | khalol |  | 3 | 300 | Foot prints found |
|  | khalol |  | 3 | 800 | one male sighted |
|  | Narh | 551 | 1 | 100 | Feacal pellets collected |
|  | Narh |  | 2 | 450 | one female sighted |
|  | Narh |  | 3 | 100 | Foot prints found |
|  | Narh |  | 3 | 100 | Feacal pellets collected |
| Kotli sattian | Badnian | 265 | 1 | 100 | Foot prints found |
|  | Badnian |  | 2 | 100 | Feacal pellets collected |
|  | Badnian |  | 2 | 100 | Foot prints found |
|  | Badnian |  | 3 | 100 | Foot prints found |
|  | Badnian |  | 3 | 100 | Feacal pellets collected |
|  | Badnian |  | 3 | 1000 | Call heard |
|  | Badnian |  | 3 | 200 | Foot prints found |
|  | Makrosh | 300 | 1 | 200 | Feacal pellets collected |
|  | Makrosh |  | 2 | 300 | Call heard |
|  | Makrosh |  | 2 | 200 | Feacal pellets collected |
|  | Makrosh |  | 2 | 200 | Foot Prints found |
|  | Makrosh |  | 2 | 200 | Foot Prints found |
|  | Makrosh |  | 3 | 100 | Call heard |
|  | Makrosh |  | 3 | 50 | Feacal pellets collected |
|  | Makrosh |  | 3 | 100 | Feacal pellets collected |
|  | Thun | 276 | 1 | 100 | Call heard |
|  | Thun |  | 1 | 400 | Foot prints found |
|  | Thun |  | 3 | 700 | male and female sighted |
|  | Thun |  | 3 | 400 | Foot prints found |
|  | Thun |  | 3 | 400 | Feacal pellets collected |
|  | Santh Sarula | 167 | 3 | 400 | Feacal pellets collected |
|  | Santh Sarula |  | 3 | 700 | Call heard |
|  | Santh Anwali | 548 | 1 | 650 | one female with one fawn sighted |
|  | Santh Anwali |  | 2 | 300 | Feacal pellets collected |
|  | Santh Anwali |  | 2 | 300 | Foot prints found |
|  | Santh Anwali |  | 3 | 300 | Feacal pellets collected |
|  | Santh Anwali |  | 3 | 100 | Call heard |
|  | Santh Anwali |  | 3 | 500 | Foot prints found |
|  | Chakka | 1567 | 1 | 500 | Feacal pellets collected |
|  | Chakka |  | 2 | 500 | Call heard |
|  | Chakka |  | 2 | 500 | Feacal pellets collected |
|  | Chakka |  | 3 | 500 | Foot prints found |
| Murree | Kathar | 1141 | 1 | 500 | Foot prints found |
|  | Kathar |  | 2 | 500 | Feacal pellets collected |
|  | Kathar |  | 2 | 500 | one female sighted |
|  | Kathar |  | 2 | 200 | one male sighted |
|  | Kathar |  | 3 | 200 | Foot prints found |
|  | Baroha | 343 | 1 | 200 | Feacal pellets collected |
|  | Baroha |  | 1 | 200 | Foot prints found |
|  | Baroha |  | 1 | 300 | call heard |
|  | Baroha |  | 2 | 200 | Feacal pellets collected |
|  | Baroha |  | 3 | 150 | Feacal pellets collected |
|  | Baroha |  | 3 | 150 | call heard |
|  | Baroha |  | 4 | 100 | one pair found |
|  | Benghal | 580 | 1 | 100 | Foot prints found |
|  | Benghal |  | 1 | 300 | one female sighted |
|  | Benghal |  | 2 | 300 | Feacal pellets collected |
|  | Benghal |  | 2 | 300 | Foot prints found |
|  | Benghal |  | 2 | 200 | call heard |
|  | Benghal |  | 3 | 300 | Feacal pellets collected |
|  | Benghal |  | 3 | 400 | Foot prints found |
|  | Benghal |  | 3 | 150 | one female sighted |
|  | Salgran | 276 | 1 | 300 | Feacal pellets collected |
|  | Salgran |  | 2 | 300 | Foot prints found |
|  | Salgran |  | 3 | 300 | Feacal pellets collected |
|  | Angoori | 149 | 2 | 300 | Feacal pellets collected |
|  | Angoori |  | 3 | 500 | call heard |
|  | Angoori |  | 3 | 700 | Feacal pellets collected |
|  | Numble | 95 | 1 | 700 | Feacal pellets collected |
|  | Numble |  | 2 | 100 | call heard |
|  | Numble |  | 3 | 700 | Foot prints found |
|  | Simli | 246 | 1 | 700 | one pair found |
|  | Simli |  | 2 | 500 | Foot prints found |
|  | Simli |  | 2 | 500 | Feacal pellets collected |
|  | Simli |  | 3 | 500 | Feacal pellets collected |
|  | Simli |  | 3 | 500 | Foot prints found |
|  | Phaphril | 287 | 1 | 500 | Feacal pellets collected |
|  | Phaphril |  | 2 | 500 | Feacal pellets collected |
|  | Phaphril |  | 2 | 1300 | one female with one fawn sighted |
|  | Phaphril |  | 3 | 100 | Foot prints found |
|  | Phaphril |  | 3 | 100 | Foot prints found |
